# Draft Genome Sequence of *Salmonella enterica* subsp. *enterica* Serovar Blockley Strain CRJJGF_00147 (Phylum *Gammaproteobacteria*)

**DOI:** 10.1128/genomeA.00954-16

**Published:** 2016-09-08

**Authors:** Sushim K. Gupta, Elizabeth A. McMillan, Charlene R. Jackson, Prerak T. Desai, Steffen Porwollik, Michael McClelland, Lari M. Hiott, Shaheen B. Humayoun, Jonathan G. Frye

**Affiliations:** a Bacterial Epidemiology and Antimicrobial Resistance Research Unit, USDA-ARS, Athens, Georgia, USA; b Department of Microbiology, University of Georgia, Athens, Georgia, USA; c Department of Microbiology and Molecular Genetics, University of California Irvine, Irvine, California, USA

## Abstract

Here, we report a 4.72-Mbp draft genome sequence of *Salmonella enterica* subsp. *enterica* serovar Blockley strain CRJJGF_00147, isolated from chicken rinse in 2009.

## GENOME ANNOUNCEMENT

*Salmonella enterica* subsp. *enterica* serovar Blockley was first isolated from the stool from a 60-year-old patient in 1955 (1). An *S*. Blockley outbreak was reported for the first time from frozen unpasteurized egg yolks used in the preparation of ice cream (2). *S*. Blockley is a rarely isolated serotype in most countries; however, *S*. Blockley has been isolated from agricultural and clinical samples periodically worldwide (3–7). Here, we announce the draft genome sequence of *S*. Blockley, isolated from chicken rinse in 2009.

Standard microbiology techniques were applied to isolate *Salmonella* strains from food animals. The isolates were serotyped using SMART typing (8), and reads were used to determine antigenic formula to predict the serotype using SeqSero (9), which predicted the antigenic formula of 8:k:1,5, designated Blockley. Using pulsed-field gel electrophoresis (PFGE), as described by PulseNet (10), the isolate was assigned PFGE pattern JBGX01.0001. Susceptibility testing for the strain was performed using broth microdilution plates for the Sensititre semiautomated antimicrobial susceptibility system (Trek Diagnostic Systems, Inc., Westlake, OH). The results were interpreted according to Clinical and Laboratory Standards Institute (CLSI) guidelines (11).

Genomic DNA was isolated from an overnight-grown culture using the GenElute bacterial genomic DNA kit (Sigma-Aldrich, St. Louis, MO). DNA libraries were constructed using Nextera-XT DNA preparation kit, and paired-end sequencing was performed on the Illumina HiSeq 2500 (Illumina, Inc., San Diego, CA) using a 500-cycle MiSeq reagent kit. A total of 2,599,790 reads were generated. Reads were *de novo* assembled using Velvet (12), which assembled to 154 contigs ≥200 bp. The combined length of contigs was 4,722,084 bases, with a G+C content of 52.11% and *N*_50_ value of 76.5 kb. The contigs were ordered with Mauve (13) using *Salmonella* LT2 as a reference genome, and coding sequences were predicted with Prodigal (14). A total of 4,399 coding sequences (≥50 amino acids) were predicted within the genome. Signal peptide, clustered regularly interspaced short palindromic repeat (CRISPR) elements, and prophages were predicted using SignalP (15), CRISPRFinder (16), and PHAST (17), respectively. We identified signal peptides in 429 coding sequences (CDSs), two CRISPR loci, and two intact phages, Gifsy2 (accession no. NC_010393) and Salmon_SEN1 (accession no. NC_029003), in the analyzed contigs. The isolate was susceptible to all tested antibiotics, although a cryptic *aac*6-Iy gene was identified through ARG-ANNOT (18). The information generated from genome sequencing will improve our understanding of the role of genomic regions and how they may lead to *Salmonella* pathogenicity.

### Accession number(s).

The genome sequence of *Salmonella enterica* subsp. *enterica* serovar Blockley strain CRJJGF_00147 has been deposited in the GenBank database (NCBI) under the accession no. JQXY00000000. This paper describes the first version of the genome.
